# Differentiation of nonhuman primate pluripotent stem cells into functional keratinocytes

**DOI:** 10.1186/s13287-017-0741-9

**Published:** 2017-12-19

**Authors:** Sophie Domingues, Yolande Masson, Aurore Marteyn, Jennifer Allouche, Anselme L. Perrier, Marc Peschanski, Cecile Martinat, Christine Baldeschi, Gilles Lemaître

**Affiliations:** 1 INSERM U-861, Institut des cellules Souches pour le Traitement et l’Etude des Maladies monogéniques (I-Stem), Association Française contre les Myopathies (AFM), 91100 Corbeil Essonnes, France; 2 Centre d’Etude des Cellules Souches (CECS), I-Stem, AFM, 91100 Corbeil Essonnes, France; 3 Université Evry Val d’Essonne (UEVE), Paris Saclay, U861, I-Stem, AFM, 91030 Evry Cedex, France

**Keywords:** Keratinocytes, Pluripotent stem cells, Nonhuman primate model, Skin graft

## Abstract

**Background:**

Epidermal grafting using cells derived from pluripotent stem cells will change the face of this side of regenerative cutaneous medicine. To date, the safety of the graft would be the major unmet deal in order to implement long-term skin grafting. In this context, experiments on large animals appear unavoidable to assess this question and possible rejection. Cellular tools for large animal models should be constructed.

**Methods:**

In this study, we generated monkey pluripotent stem cell-derived keratinocytes and evaluated their capacities to reconstruct an epidermis, in vitro as well as in vivo.

**Results:**

Monkey pluripotent stem cells were differentiated efficiently into keratinocytes able to reconstruct fully epidermis presenting a low level of major histocompatibility complex class-I antigens, opening the way for autologous or allogeneic epidermal long-term grafting.

**Conclusions:**

Functional keratinocytes generated from nonhuman primate embryonic stem cells and induced pluripotent stem cells reproduce an in-vitro and in-vivo stratified epidermis. These monkey skin grafts will be considered to model autologous or allogeneic epidermal grafting using either embryonic stem cells or induced pluripotent stem cells. This graft model will allow us to further investigate the safety, efficacy and immunogenicity of nonhuman primate PSC-derived epidermis in the perspective of human skin cell therapy.

**Electronic supplementary material:**

The online version of this article (doi:10.1186/s13287-017-0741-9) contains supplementary material, which is available to authorized users.

## Background

Preclinical models for regenerative medicine require the use of large animals possessing similar physiology and immunological response to human. In this context, nonhuman primates have a unique role in translational preclinical studies using pluripotent stem cell derivatives. Extensive skin loss and chronic wounds are still a significant challenge to clinicians mainly due to the fact that skin surgical procedures are often limited by the poor ability of healthy tissue [1]. Based on their ability for self-renewal and differentiation into most cell types of the organism, human pluripotent stem cells therefore offer a relevant opportunity to address this issue. We demonstrated previously that human embryonic stem cells can be differentiated efficiently into keratinocytes giving rise to a pluristratified epidermis graftable onto immunodeficient mice [2]. These results were recently extended to human induced pluripotent stem cells, demonstrating the promise of this differentiation protocol for skin graft regenerative medicine applications [3, 4]. Nonetheless, the demonstration of the therapeutic potential offered by pluripotent stem cell-derived reconstructed epidermis resides in the evaluation of this therapeutic product within large animal models since several studies support the importance of such animal models for clinical translation of pluripotent-derived cellular therapy [5–7].

The present study concerns the first development of nonhuman primate pluripotent stem cell-derived reconstructed epidermis that opens new perspectives to evaluate the efficacy and safety of skin graft in preclinical studies.

## Methods

### Cell culture

Fibroblasts were isolated from monkey (*Macaca fascicularis*) skin biopsy. Primary monkey fibroblasts and the human (DM4603; Coriell) cell line were successfully reprogrammed into iPSCs using retroviral vector harboring OCT4, KLF4, SOX2, c-MYC [8]. Monkey ESCs [9] (*Macaca mullata*, ORMES 18) and iPSCs [10] as well as hESC SA001 (Cellartis, Gothenburg, Sweden), and hiPSCs were cultured as described previously [2, 9, 10]. PSCs were seeded on mitomycin C-treated mouse embryonic fibroblast (MC-MEF) in ESC medium (ESM; DMEM/F12 supplemented with 20% KO serum replacement, 1% nonessential amino acid, and 10 ng/ml basic FGF (Peprotech®)). Colonies of PSCs were mechanically picked up and replaced on MC-MEF for expansion and characterization.

### Keratinocyte differentiation

**Fig. 1:**
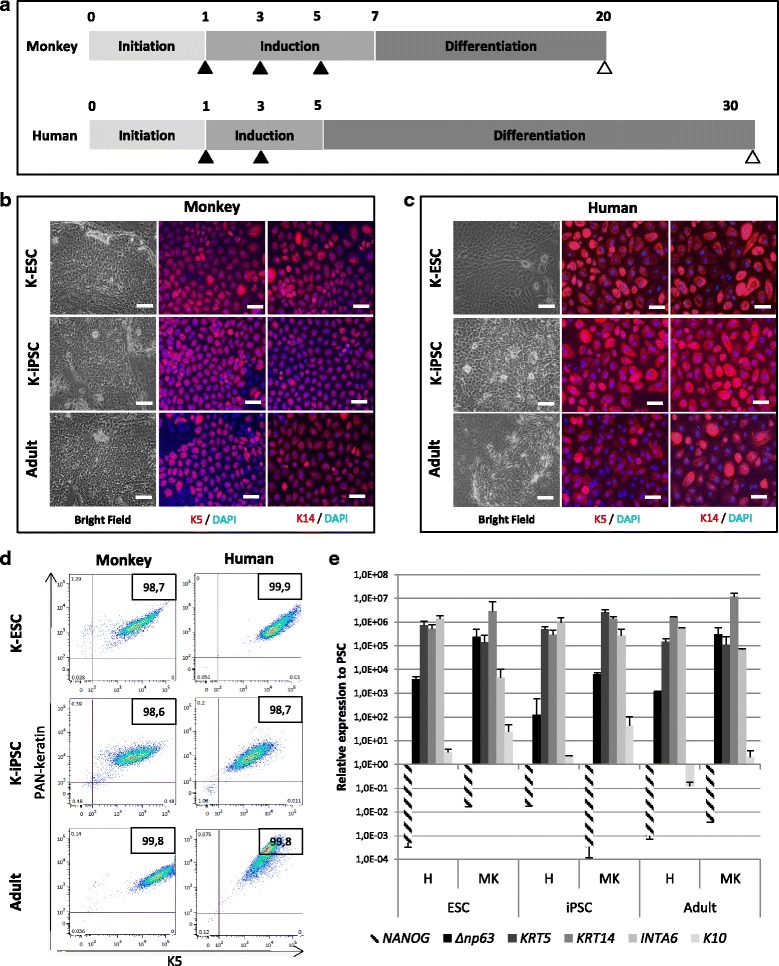
Differentiation and characterization of monkey keratinocytes derived from PSCs. **a** Schematic of protocol design for differentiation of monkey and human pluripotent stem cells into keratinocytes. Black triangles represent medium change (containing RA and BMP4) and white triangles depict end of differentiation. **b** Monkey and **c** human K-PSC phase-contrast microscopy analysis (scale bar: 100 μm) and immunofluorescence analysis for keratinocyte markers: keratin 5 (K5) and keratin 14 (K14) (scale bar: 50 μm). Adult keratinocytes represented as control. **d** Representative fluorescence-activated cell sorting analysis. Keratinocyte suspensions were fixed, permeabilized and stained with K5 and a panel of cytokeratin 14, 15, 16 and 19 (PAN-keratin) in keratinocytes derived from pluripotent stem cells and adult keratinocytes as biological control. Isotype-specific antibodies used as control. Cells analyzed on a MACSQuant Analyzers system (10,000 events per experiment). **e** Quantitative PCR analysis of *NANOG*, *ΔNp63*, *KRT5*, *KRT14*, *INTA6* and *KRT10* in monkey and human K-PSCs and adult keratinocytes. DAPI 4′,6-diamidino-2-phenylindole, ESC embryonic stem cells, H human, iPSC induced pluripotent stem cells, K-ESC keratinocytes derived from embryonic stem cells, K-iPSC keratinocytes derived from induced pluripotent stem cells, M monkey

Differentiation protocols for the two species are presented in Fig. 1a. Briefly, monkey and human PSCs were seeded for 24 h on Matrigel™ (BD Biosciences)-coated plates in ESM. The medium was then replaced with FAD medium, as described by Guenou et al. [2], supplemented with retinoic acid (RA) (1 μM; Sigma Aldrich®) and BMP4 (0.05 nM; Peprotech®) during 6 days for monkey cells (medium change at D1, D3 and D5 represented by black triangles in the figure) and with defined-KSFM® (ThermoFisher Scientific) supplemented with RA (1 μM) and BMP4 (0.5 nM) during 4 days for human cells (medium change at D1 and D3 represented by black triangles). On D7 for monkey or D5 for human, the medium was replaced by the defined-KSFM® medium, until D20 for monkey or D30 for human (differentiation ending is represented by white triangles). Both monkey and human keratinocytes were amplified in keratinocyte medium (CnT07; CELLnTEC) on collagen type I-coated plates (Biocoat; BD Biosciences).

### RT-qPCR

Total RNA was extracted with RNeasy Mini extraction kit (Qiagen) according to the manufacturer’s protocol. Gene expression quantification was based on the ΔCt method, normalized with 18S expression. Melting curves and electrophoresis analyses were done to control specificities of PCR products and to exclude nonspecific amplification.

### In-vitro and in-vivo reconstructed epidermis

Organotypic epidermis was generated as described previously [11] on polycarbonate culture inserts (Millipore). Tissues were generated using keratinocytes (passage 1) maintained in CnT07 medium in immersion during 24 h to allow cell attachment on the membrane. The medium was switched to a reconstruction medium (CnT02; CELLnTEC) during 24 h. Finally, keratinocytes were placed at the air–liquid interface for 14 days, to allow stratification.

Plasma-based fibrins were prepared at 600 μl/cm^2^ (10 cm^2^ per mouse) and monkey K-iPSCs and primary keratinocytes were seeded at 150,000 cells/cm^2^. Generated tissues were cultured using defined-KSFM® during 8 days with a pulse of 0.39 mg/ml Exacyl, 4 days after seeding. On the day of grafting, 6-week-old male nude mice (Crl:NU(Ico)-Fox1nu; Charles River Laboratories, France) were firstly anesthetized with a combination of inhaled isoflurane (Isoflurane 1000 mg/g; Iso-Vet, Eurovet)/O_2_ (5%/95%) then the concentration of isoflurane was reduced (3 ± 0.5%) and adapted depending on mouse size and behavior during the procedure (2 mice per keratinocyte type). Skin windows of 4 cm^2^ were cut on mice’s backs and devitalized by five heating/thawing cycles with liquid nitrogen and PBS 1× bathes. The generated tissues were then placed on the cut area and covered with flexible lipido colloid contact layer (UrgoTül; URGO). The devitalized tissues were sutured with remaining skin to protect the grafting area. After 2 weeks of grafting, tissues were harvested from the animal under sedation using a combination of intraperitoneal Ketamine® (60 mg/kg; Virbac France) and xylazine (7 mg/kg; Rompun®; Bayer Healthcare). All animals were then sacrificed with an overdose of anesthetics, according to the French institutional animal guidelines.

### Statistical analysis

Three independent experiments were performed for each cell line for differentiation, organotypic epidermis, RT-qPCR, immunocytochemistry and FACS analysis.

## Results

In order to evaluate the ability of Macaca monkey pluripotent stem cells (monkey PSCs) to give rise to pluristratified epidermis, monkey embryonic stem cells (ORMES 18) and monkey induced pluripotent stem cells were compared to their human counterparts. At the undifferentiated level, immunofluorescence and qPCR analyses (Additional file 1) showed that monkey PSCs express typical pluripotent markers to human PSCs: *OCT4*, *NANOG*, *SOX2*, *KLF4* and *cMYC* (for primer sequences, see Additional file 2). As expected, no expression of the differentiated markers was observed (Additional file 1). G-banding analysis was performed on monkey and human PSCs, showing no abnormalities on their karyotypes: 42,XY for monkey PSC lines and 46,XY for human PSC lines (Additional file 1).

Based on recent protocols using human pluripotent stem cells [2, 3], we evaluated the capacity of monkey PSCs to be converted into functional keratinocytes. Pluripotent stem cells were seeded on a Matrigel™ matrix and differentiation was induce by treating cells with medium supplemented with retinoic acid and BMP4 (black triangles in Fig. 1a). After this induction phase, monkey and human cells were placed in defined-KSFM® medium. Interestingly, at the morphological level, monkey keratinocyte-like cells were observed as early as 20 days of differentiation compared to the 30 days of differentiation necessary for human PSCs (white triangles in Fig. 1a). At the end of the differentiation phase, keratinocytes were amplified on a Collagen I Biocoat plastic in CnT07 medium. Confirming this observation, the expression level of keratinocyte markers at 20 days of differentiation for monkey PSCs achieved a comparable level to that observed with human PSCs at 30 days of differentiation. Immunofluorescence analysis showed that basal keratins K5 and K14 (Fig. 1b, c) were expressed similarly in the cytoplasm between the two species. FACS analysis demonstrated that a homogeneous keratinocyte population, presenting double-positive specific keratin labeling (>98.5% in all cases), was similar whatever the cell origin and species (Fig. 1d). By RT-qPCR analysis, similar profiles of selected gene expression were observed between all keratinocytes derived from PSCs (K-PSCs) and were similar to the control adult keratinocytes (Fig. 1e). Basal keratinocyte markers *ΔNp63*, *KRT5*, *KRT14* and *INTA6* were highly expressed whereas *NANOG* was underexpressed and the late epidermal marker *KRT10* was low expressed.

**Fig. 2:**
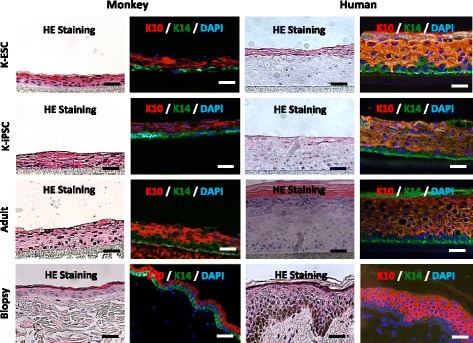
In-vitro reconstruction of epidermis using monkey and human keratinocytes derived from pluripotent stem cells. Organotypic epidermis was generated using keratinocytes at passage 1. Hematoxylin–eosin (HE) staining of monkey and human K-ESC and K-iPSC reconstructed epidermis, organotypic cultures of primary keratinocytes and skin biopsies, (scale bar: 50 μm) and analysis of the expression and location of keratin 10 (K10) and keratin 14 (K14) (scale bar: 20 μm) in monkey and human K-ESC and K-iPSC reconstructed epidermis, organotypic cultures of primary keratinocytes and skin biopsies. DAPI 4′,6-diamidino-2-phenylindole, K-ESC keratinocytes derived from embryonic stem cells, K-iPSC keratinocytes derived from induced pluripotent stem cells

To evaluate the functionality of monkey PSC-derived keratinocytes, their ability to form an in-vitro reconstructed epidermis was analyzed. Monkey K-PSCs were capable of forming an epidermis with three to four layers similar to the monkey skin biopsy (Fig. 2a). As expected, monkey epidermis is thinner than human epidermis. The latter present four defined layers (basal, spinous, granular and corneal layers) while in the monkey epidermis a rapid transition from the basal layer to the corneal layer separated by an intermediate spinous/granular layer was observed. Expression of keratin 14 in the basal layer and keratin 10 on suprabasal layers (Fig. 2b) was observed in reconstructed epidermis with adult keratinocytes and K-PSCs for the two species, indicating a normal stratification of the reconstructed epidermis.

**Fig. 3:**
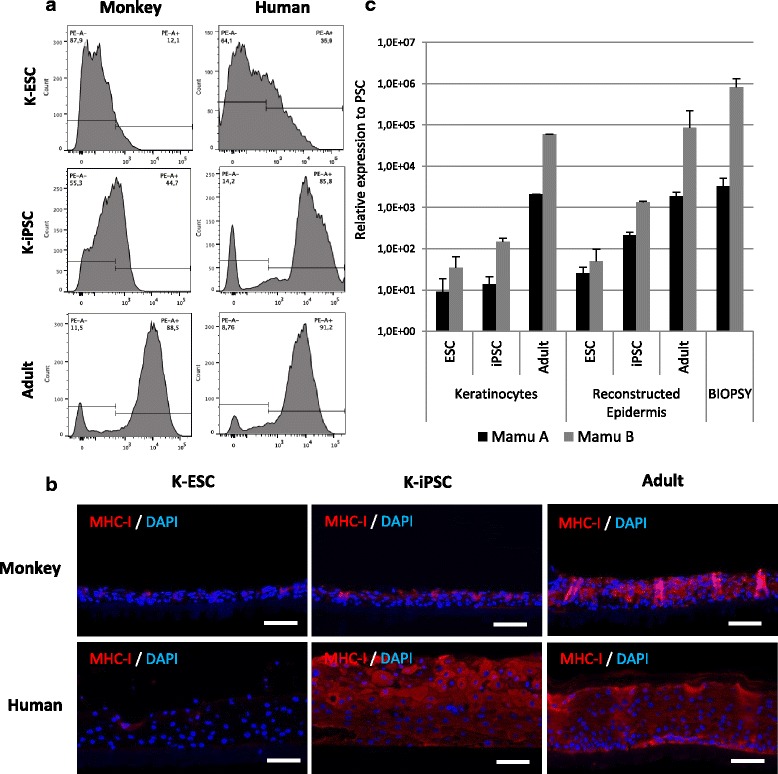
Expression of MHC-I antigens in human and monkey PSC-derived keratinocytes and epidermis. **a** Representative fluorescence-activated cell sorting analysis of major histocompatibility complex class I (MHC-I) in monkey and human keratinocytes derived from ESCs and iPSCs and in adult keratinocytes. **b** Immunofluorescence analysis of the expression of MHC-I antigens in in-vitro reconstructed monkey and human epidermis from K-ESCs, K-iPSCs (at passage 1) and adult keratinocytes (scale bar: 20 μm). **c** TaqMan gene expression assay by qPCR analysis of monkey specific MHC-I genes: Mamu A and Mamu B in monkey keratinocytes derived from pluripotent stem cells and in-vitro reconstructed epidermis (at passage 1). Results expressed as relative expression to monkey PSCs. DAPI 4′,6-diamidino-2-phenylindole, ESC embryonic stem cell, iPSC induced pluripotent stem cell, K-ESC keratinocytes derived from embryonic stem cells, K-iPSC keratinocytes derived from induced pluripotent stem cells

We next sought to quantify the expression of major histocompatibility complex class I (MHC-I) in keratinocytes derived from monkey and human PSCs using flow cytometry, immunostaining and RT-qPCR analyses (Fig. 3). Monkey and human K-ESCs expressed relatively low levels of MHC-I antigens (12.1% and 35.9% respectively). Concerning K-iPSCs, monkey cells express less MHC-I (44.7%) than human cells (85.6%). Adult keratinocytes expressed a high level of those antigens (more than 85%) in both species. The expression of MHC-I was also analyzed by immunofluorescence in the reconstructed epidermis (Fig. 3b). Using adult keratinocytes for both species or K-hiPSCs, MHC-I antigens were located in all layers of the epidermis, while in reconstructed epidermis with monkey K-ESCs and K-iPSCs and human K-ESCs, only a few cells on the upper layers express MHC-I antigens. The expression of Mamu A and Mamu B antigens corresponding to genes coding for the two monkey MHC-I antigen families were analyzed by RT-qPCR in monkey cells (Fig. 3c). In keratinocytes, the expression of Mamu antigens was quite similar between K-PSCs. In comparison, adult keratinocytes express higher levels of Mamu antigens than K-PSCs.

**Fig. 4:**
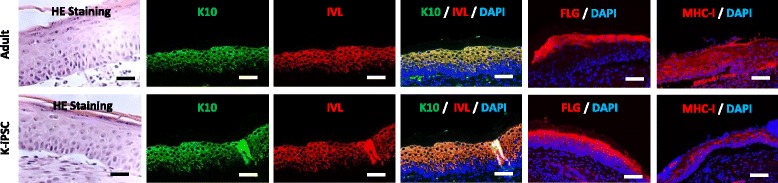
Short-term in-vivo monkey epidermal reconstruction following xenografting to nude mice. Hematoxylin–eosin (HE) staining of monkey K-iPSC and primary keratinocyte epidermis after 14 days of grafting (scale bar: 50 μm) and analysis of the expression and location of keratin 10 (K10), involucrin (IVL), filagrin (FLG) and major histocompatibility complex class I (MHC-I) proteins (scale bar: 20 μm). DAPI 4′,6-diamidino-2-phenylindole, K-iPSC keratinocytes derived from induced pluripotent stem cells

Finally, keratinocytes derived from monkey adult or iPSCs were seeded on a fibrin matrix and graft on the back of nude mice. Fourteen days after grafting, the graft epidermis was analyzed by immunohistochemistry. A pluristratified epidermis was present and visualized by HE staining (Fig. 4), presenting all the layers of the epidermis including the horny layer. All classical markers of keratinocyte differentiation were present and well located in this epidermis. Moreover, as for in-vitro reconstruction, the expression of the species-specific MHC-I antigens was restricted more in the upper layers in the iPSC-derived epidermal graft.

## Discussion

This study describes for the first time the possibility to efficiently and rapidly differentiate nonhuman primate PSCs into functional keratinocytes.

Interestingly, our results highlight the fact that this rapid differentiation process occurs with the same developmental cue molecules that are required for mammal development [2, 12]. The monkey K-ESCs and K-iPSCs have the ability to form a multilayered epidermis in vitro and in vivo resembling an adult monkey epidermis. These cells represent a new tool to progress toward clinical application. The reconstructed epidermis using keratinocytes derived from pluripotent stem cells could serve as a skin substitute in a wound healing surface such as ulcers [13]. Because of their early developmental state, the human K-ESCs express a low level of MHC-I antigens [2], as has been also demonstrated in this study using monkey PSC-derived keratinocyte and epidermis, suggesting a low immunogenicity of the skin substitute. The establishment of a cellular tool will permit future assays of the immune rejection of an epidermal graft derived from PSCs during allogeneic or autologous administration.

## Conclusion

Altogether, these results clearly pave the way for the possibility of using these keratinocytes and reconstructed epidermis products for clinical investigations within a large animal model.

## Additional files

Additional file 1: A figure showing characterization of monkey and human pluripotent stem cells. (**A**) Immunofluorescence analysis of monkey PSCs compared to human PSCs. Cells fixed in 4% paraformaldehyde before permeabilization and blocking in phosphate buffer solution supplemented with 0.1% Triton and 5% bovine serum albumin. Primary antibodies incubated overnight at 4 °C in blocking buffer: mouse anti Oct3/4 and rabbit anti Nanog (scale bar: 50 μm). (**B**) Gene expression profiles analyzed by RT-qPCR analysis for pluripotency markers *cMYC*, *KLF4*, *OCT4*, *SOX2* and *NANOG*, and the three germ layer markers *AFP*, *CDX2*, *GATA4*, *GATA6*, *NESTIN* and *KRT5* in pluripotent stem cells of the two species (black bars for monkey, gray bars for human). Results expressed as relative expression to human teratocarcinoma cDNA. (**C**) G-Banding karyotype of monkey and human PSCs. (PDF 348 kb)

Additional file 2: A table presenting Syber GREEN quantitative PCR primer sequences. (PDF 48 kb)

## Abbreviations

BMP4: Bone morphogenic protein
ESC: Embryonic stem cell
ESM: ESC medium
FACS: Fluorescent-activated cell sorting
FGF: Fibroblast growth factor
HE: Hematoxylin–eosin
iPSC: Induced pluripotent stem cell
K-PSC: Keratinocyte derived from pluripotent stem cell
KSFM: Keratinocyte serum free medium
MC-MEF: Mitomycin C-treated mouse embryonic fibroblast
MHC-I: Major histocompatibility complex class I
PBS: Phosphate buffer saline
PSC: Pluripotent stem cell
RA: Retinoic acid
RT-qPCR: Reverse transcriptase quantitative polymerase chain reaction

## References

[1] Sun BK, Siprashvili Z, Khavari PA (2014). Advances in skin grafting and treatment of cutaneous wounds. Science.

[2] Guenou H, Nissan X, Larcher F (2009). Human embryonic stem-cell derivatives for full reconstruction of the pluristratified epidermis: a preclinical study. Lancet.

[3] Itoh M, Kiuru M, Cairo MS (2011). Generation of keratinocytes from normal and recessive dystrophic epidermolysis bullosa-induced pluripotent stem cells. Proc Natl Acad Sci U S A.

[4] Itoh M, Umegaki-Arao N, Guo Z (2013). Generation of 3D skin equivalents fully reconstituted from human induced pluripotent stem cells (iPSCs). PLoS One.

[5] Blin G, Nury D, Stefanovic S (2010). A purified population of multipotent cardiovascular progenitors derived from primate pluripotent stem cells engrafts in postmyocardial infarcted nonhuman primates. J Clin Invest.

[6] Okamoto S, Takahashi M (2011). Induction of retinal pigment epithelial cells from monkey iPS cells. Invest Ophthalmol Vis Sci.

[7] Zhu FF, Zhang PB, Zhang DH (2011). Generation of pancreatic insulin-producing cells from rhesus monkey induced pluripotent stem cells. Diabetologia.

[8] Takahashi K, Tanabe K, Ohnuki M (2007). Induction of pluripotent stem cells from adult human fibroblasts by defined factors. Cell.

[9] Thomson JA, Kalishman J, Golos TG (1995). Isolation of a primate embryonic stem cell line. Proc Natl Acad Sci U S A.

[10] Liu H, Zhu F, Yong J (2008). Generation of induced pluripotent stem cells from adult rhesus monkey fibroblasts. Cell Stem Cell.

[11] Poumay Y, Dupont F, Marcoux S (2004). A simple reconstructed human epidermis: preparation of the culture model and utilization in in vitro studies. Arch Dermatol Res.

[12] Coraux C, Hilmi C, Rouleau M (2003). Reconstituted skin from murine embryonic stem cells. Curr Biol.

[13] Lemaitre G, Nissan X, Baldeschi C (2011). Concise review: Epidermal grafting: the case for pluripotent stem cells. Stem Cells.

