# Supplementary figures and images for: Differentiation of nonhuman primate pluripotent stem cells into functional keratinocytes
Source: Stem Cell Res Ther. 2017 Dec 19;8:285. doi: 10.1186/s13287-017-0741-9 (PMC5738144; doi:10.1186/s13287-017-0741-9)

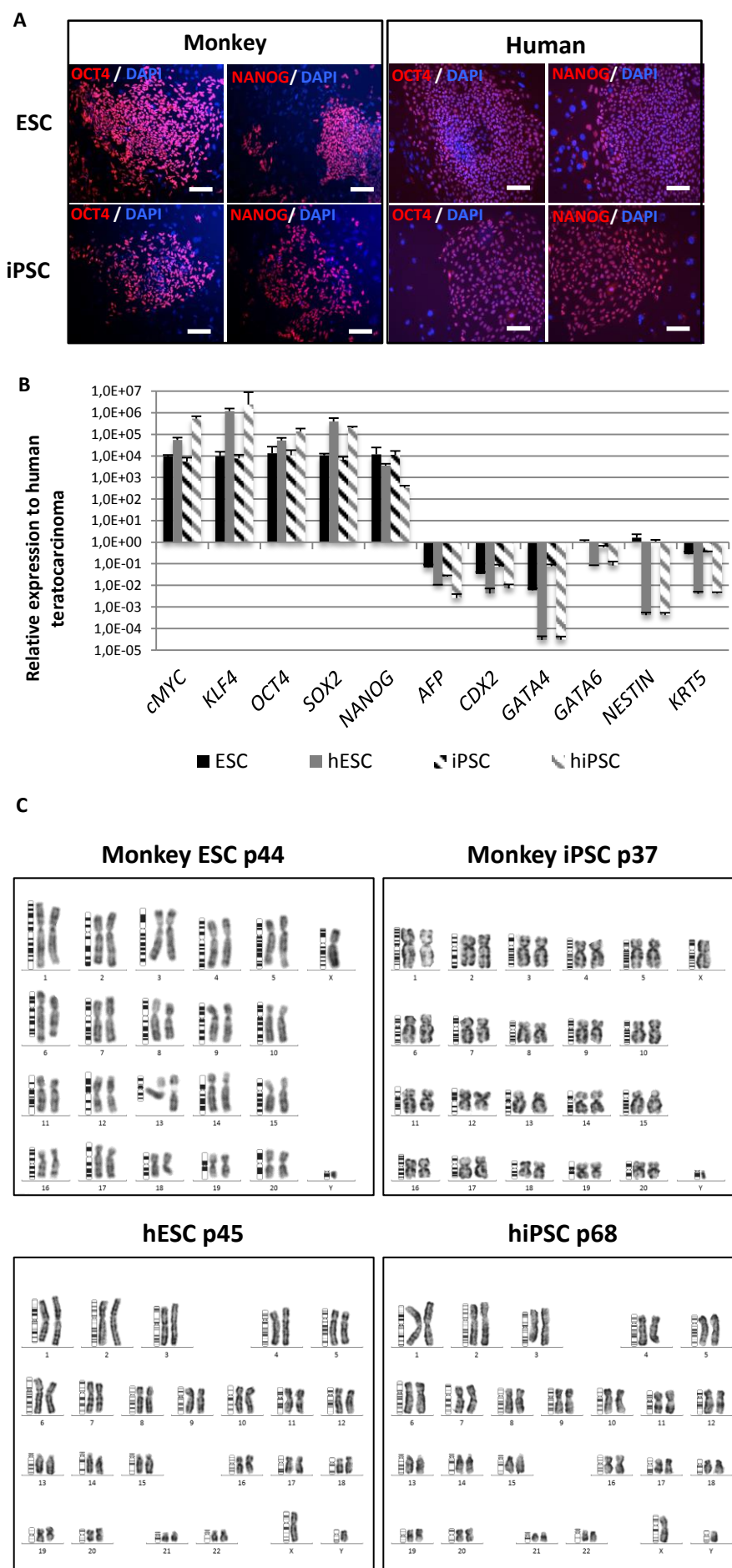

**Figure S1 : Characterization of monkey and human pluripotent stem cells.**

Supplement: Supplementary file 1 — A figure showing characterization of monkey and human pluripotent stem cells. (A) Immunofluorescence analysis of monkey PSCs compared to human PSCs. Cells fixed in 4% paraformaldehyde before permeabilization and blocking in phosphate buffer solution supplemented with 0.1% Triton and 5% bovine serum albumin. Primary antibodies incubated overnight at 4 °C in blocking buffer: mouse anti Oct3/4 and rabbit anti Nanog (scale bar: 50 μm). (B) Gene expression profiles analyzed by RT-qPCR analysis for pluripotency markers cMYC, KLF4, OCT4, SOX2 and NANOG, and the three germ layer markers AFP, CDX2, GATA4, GATA6, NESTIN and KRT5 in pluripotent stem cells of the two species (black bars for monkey, gray bars for human). Results expressed as relative expression to human teratocarcinoma cDNA. (C) G-Banding karyotype of monkey and human PSCs. (PDF 348 kb) [file 13287_2017_741_MOESM1_ESM.pdf]
