# Supplementary material for: Differentiation of nonhuman primate pluripotent stem cells into functional keratinocytes
Source: Stem Cell Res Ther. 2017 Dec 19;8:285. doi: 10.1186/s13287-017-0741-9 (PMC5738144; doi:10.1186/s13287-017-0741-9)
Supplement: Supplementary file 2 — A table presenting Syber GREEN quantitative PCR primer sequences. (PDF 48 kb) [file 13287_2017_741_MOESM2_ESM.pdf]

Additional file 2 : Syber GREEN quantitative PCR primers sequences

Primers sequences	Target genes
GAGGATGAGGTGGAACGTGT	18S F
TCTTCAGTCGCTCCAGGTCT	18S R
ACTCTGAGGAGGAACAAGAA	cMYC F
TGGAGACGTGGCACCTCTT	cMYC R
TCTCAAGGCACACCTGCGAA	KLF4 F
TAGTGCCTGGTCAGTTCATC	KLF4 R
CTTGCTGCAGAAGTGGGTGGAGGAA	OCT 4 F
CTGCAGTGTGGGTTTCGGGCA	OCT 4 R
AGCTACAGCATGATGCAGGA	SOX2 F
GGTCATGGAGTTGTACTGCA	SOX2 R
CAAAGGCAAACAACCCACTT	NANOG Human F
TCTGCTGGAGGCTGAGGTAT	NANOG Human R
TGCCTGGTGAACCCGACTGGGA	NANOG Monkey F
GGCTGCTCCAGGGTGGGACG	NANOG Monkey R
TGCCAACTCAGTGAGGACAA	AFP F
TCCAACAGGCCTGAGAAATC	AFP R
TCAGCCAGGTCCTCTGAGAA	CDX2 F
GCCTGGAATTGCTCTGCC	CDX2 R
GCCTCTACATGAAGCTCCA	GATA4 F
GGCTGTTCCAAGAGTCCTGC	GATA4 R
GCCAACTGTCACACCACAAC	GATA6 F
ACGCCTATGTAGAGCCCATC	GATA6 R
TGGGAAAGGACCAAGAACTG	NESTIN F
TCAGCTAGGCCTCTCTGCTC	NESTIN R
ATCTCTGAGATGAACCGGATGATC	KRT5 F
CAGATTGGCGCACTGTTTCTT	KRT5 R
GGCCTGCTGAGATCAAAGACTAC	KRT14 F
CACTGTGGCTGTGAGAATCTTGTT	KRT14 R
TGGTTCAATGAAAAGAGCAAGGA	KRT10 F
GGGATTGTTTCAAGGCCAGTT	KRT10 R
GGAAAACAATGCCCAGACTC	ΔnP63 F
GTGGAATACGTCCAGGTGGC	ΔnP63 R
GCTGGTTATAATCCTTCAATATCAATTGT	ITGA6 F
TTGGGCTCAGAACCTTGGTTT	ITGA6 R
